# Provision of guideline-based care for drug-resistant tuberculosis in South Africa: Level of concordance between prescribing practices and guidelines

**DOI:** 10.1371/journal.pone.0203749

**Published:** 2018-11-05

**Authors:** Brittney J. van de Water, Susan G. Silva, Janet Prvu Bettger, Janice Humphreys, Coleen K. Cunningham, Jason E. Farley

**Affiliations:** 1 Duke University School of Nursing, Duke University, Durham, NC, United States of America; 2 Duke Global Health Institute, Duke University, Durham, NC, United States of America; 3 Department of Global Health and Social Medicine, Harvard Medical School, Boston, MA, United States of America; 4 Department of Orthopaedic Surgery, Duke University School of Medicine, Durham, NC, United States of America; 5 Department of Pediatrics, Duke University School of Medicine, Durham, NC, United States of America; 6 Department of Community Public Health, Johns Hopkins University School of Nursing, Baltimore, MD, United States of America; University of Cape Town, SOUTH AFRICA

## Abstract

**Title:**

Provision of guideline-based care for drug-resistant tuberculosis in South Africa: Level of concordance between prescribing practices and guidelines.

**Objective:**

We examined the influence of individual and site characteristics on the concordance between prescribed treatment regimens and recommended standardized regimen according to national guidelines for patients with drug-resistant tuberculosis (DR-TB) in South Africa.

**Methods:**

Participants were 337 youth and adults treated for DR-TB between November 2014 and August 2016 at ten DR-TB treatment sites in Eastern Cape and KwaZulu Natal provinces, South Africa. Logistic regression was used to determine individual and system characteristics related to concordance at treatment initiation between the prescribed treatment regimens in terms of medication composition, dosage, and frequency and guideline-based standardized regimen that included four oral and one injectable medications.

**Results:**

The sample was 19% (n = 64) youth (15–24 years), 53% (n = 179) male, 73% (n = 243) HIV coinfected, and 51% (n = 169) with prior history of TB treatment. Guideline medications were correctly prescribed for 88% (n = 295) of patients, but only 33% (n = 103) received the correct medications and doses. Complete guideline adherence to medications, doses, and frequency was achieved for 30% (n = 95) of patients. Younger age, HIV coinfection, and rural treatment setting were associated with the prescription of correct medications.

**Conclusion:**

Most individuals are prescribed the correct DR-TB medications, yet few individuals receive correct medications, dosages, and frequencies. Further study is needed to examine the root causes for treatment guideline deviations and opportunities for improvement.

## Introduction

Only 50% of individuals are successfully treated globally for drug-resistant *Mycobacterium tuberculosis* (DR-TB) [1]. Patients should be linked with high quality TB programs to successfully treat DR-TB [1]. Thus, providers must be aware of guidelines to appropriately scale up DR-TB services. Poor adherence to guidelines at the healthcare system-level causes DR-TB to spread [2, 3].

As of 2016 in South Africa, only 59% of individuals diagnosed with DR-TB initiate treatment within the same year [4]. Of those, only half successfully complete treatment or are cured [4]. Although DR-TB treatment is more difficult to treat, it is treatable and curable with second-line medications [5]. The South African National Department of Health disseminated treatment guidelines in 2013 [5], yet provision of guideline-based care for DR-TB is unknown.

Studies have shown that youth 15 to 24 years are exceptionally vulnerable to delays in diagnosis, treatment initiation, and appropriate treatment of HIV [6–11], yet there is a paucity of research pertaining to DR-TB treatment in youth [12, 13]. The purpose of this secondary analysis was to examined the influence of individual and site characteristic factors on the concordance at treatment initiation between the prescribed treatment regimens in terms of medication composition, dosage, and frequency and recommended standardized regimen according to national guidelines for patients with DR-TB in South Africa.

## Methods

### Design

This secondary analysis examined data from a 5-year cluster randomized trial investigating a nurse case management (NCM) intervention in individuals older than 13 years with DR-TB (R01 AI104488-01A1; PI: Farley). The analysis was designed to retrospectively determine whether patients with DR-TB received the correct combination of medicines, at the correct doses, and correct frequencies at treatment initiation (*regimen*), and identify individual and site characteristics associated with prescription of the correct regimen as per the South African DR-TB guideline recommended care. Institutional Review Board approval for this secondary analysis was provided by Duke University (Pro00067846) and Johns Hopkins University (NA_00078899/CIR00009135). An amendment to parent study, including this sub-study, was reviewed and approved by the Provincial Health Research Committees of the KwaZulu-Natal and Eastern Cape Provincial Department of Health, and the parent study was approved by the Biomedical Research and Ethics Committee of the University of KwaZulu-Natal and the Institutional Review Board of the Johns Hopkins Medical Institutions. Informed consent was provided by participants/guardians per the parent study. Required permits and approvals for foreign researchers were obtained.

#### Parent study

The parent trial began in November 2014. The study includes 10 sites randomized to a NCM intervention or observational control. The parent intervention consists of a nurse coordinating treatment with weekly phone calls or visits during the intensive six months of treatment and monthly visits during the 30-month continuation phase. Patients are excluded if enrolled in other trials. The parent study and pilot intervention [14] are described elsewhere. All 10 sites were used in this study as initial DR-TB regimen prescribing occurred by non-study clinicians prior to the intervention. Thus the NCM intervention had no influence on initial regimen prescribing concordance, nor the type of prescriber initiating therapy. Nurse case managers did not prescribe therapy, but worked with a prescribing clinician to offer supportive services to patients post treatment initiation.

#### Setting and sample

**Fig 1:**
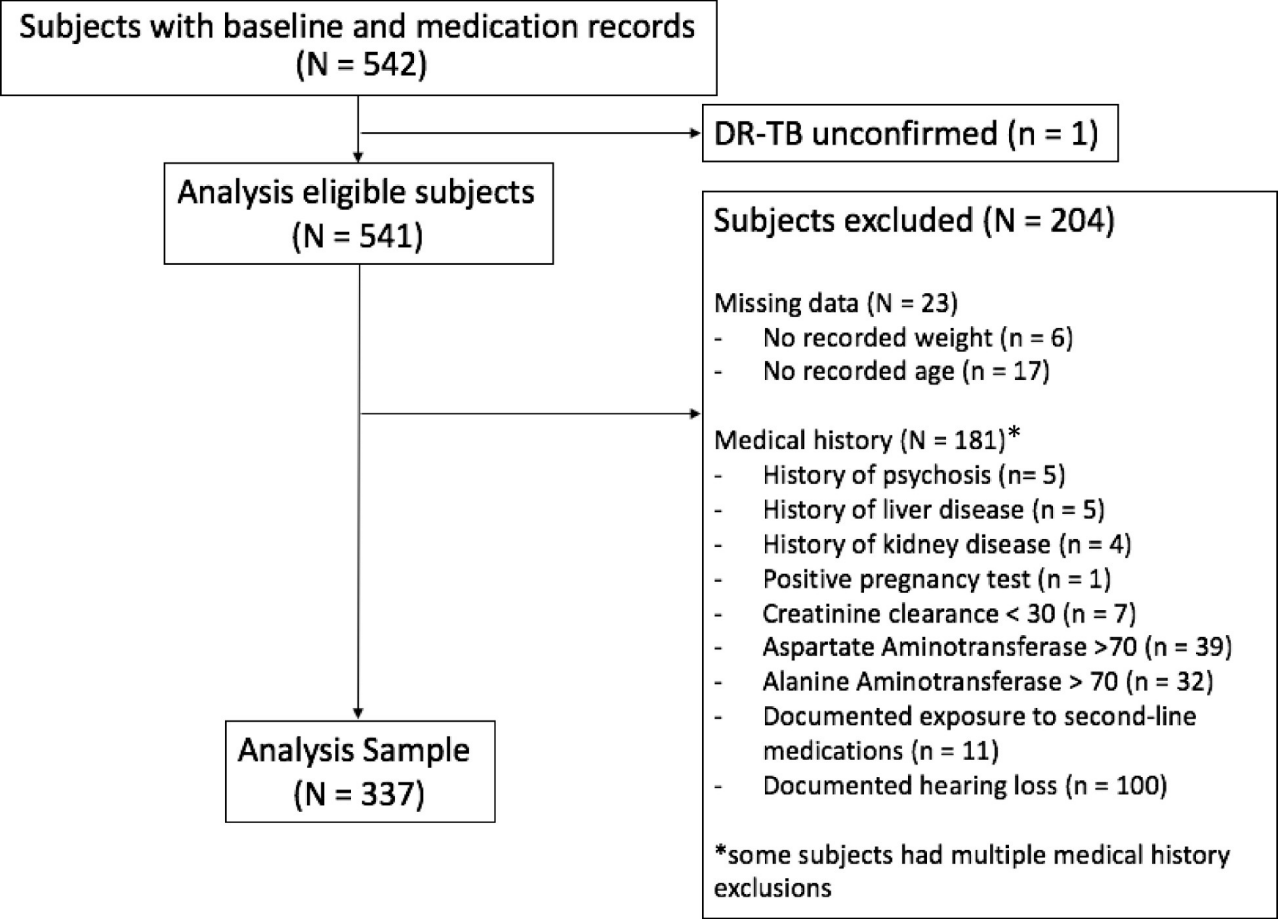
Participant flow chart.

Data were from patients at 10 department of health hospitals, offering free care in KwaZulu Natal (KZN) and the Eastern Cape (EC), two provinces with high DR-TB burdens [5]. The majority of individuals receiving care are Black South Africans. Individuals with DR-TB initiating treatment between November 2014 and August 2016 were eligible. Of 542 patients with data available, 205 were unconfirmed for DR-TB, or were clinically contraindicated for standard DR-TB regimens [5]. Standardized regimens are known to cause liver and kidney damage, ototoxicity, exacerbate psychiatric conditions, and are contraindicated in pregnancy [15, 16]. Patients were excluded from the analysis if they had: history of liver disease, aspartate aminotransferase (AST), or alanine aminotransferase (ALT) >70 (twice the upper limit of normal); history of kidney disease or creatinine clearance (CrCl) <30 (South African definition of impaired renal function); history of psychosis; any confirmed hearing loss (>20 decibels (dB)); positive pregnancy test; history of receiving second-line DR-TB medication. Thus, a total of 337 participants without known clinical indications to modify standardized regimens were included (Fig 1).

#### Data sources and procedures

Data were stored in binders by study personnel. After quality assurance comparing medical records with case report forms, forms were scanned and manually entered into REDCap, a web-based application. Data were downloaded into an analysis dataset and archived on a secure server. Original forms were checked to determine accuracy of the data entered into REDCap. Less than 0.5% of data were incorrectly coded when 10% of charts were checked. Data analysis was conducted using SAS (version 9.3, Cary, NC).

### Measures

#### Guideline adherence outcomes: Prescription of correct DR-TB regimen

The term “guideline adherence” was used to represent the concordance at treatment initiation between the prescribed treatment regimens in terms of medication composition, dosage, and frequency and recommended standardized regimen according to national guidelines.

DR-TB medications prescribed at treatment initiation refers to the first medications prescribed after study enrollment as well as within two days of the first prescribed medication. Standardized treatment was expected as patients began treatment prior to drug susceptibility testing (DST) results and was based upon GeneXpert diagnosis.

Guideline adherence to the South African guideline for DR-TB treatment after study enrollment and during treatment initiation was the primary outcome. To determine adherence, each *regimen* was compared to the standardized South African DR-TB treatment including at least four oral medications: Moxifloxacin, Ethionamide, Pyrazinamide, Terizidone, and one injectable medication: Kanamycin, Amikacin, or Capreomycin.

Adherence to *regimen* required meeting all of the following criteria: a) prescription of all five standardized medications; b) correct weight-based dosage; c) given at the recommended frequency (S1 Table). South Africa guidelines state that all medications should be administered at least six days per week. However, injectable agents given five days per week was accepted as adherent as many health centers in South Africa only give injections on week days due to human resource constraints. Additional outcomes included subcomponents of the *regimen* prescribed during treatment initiation, and included (1) prescription of the recommended combination of oral and injectable medications, and (2) prescription of the recommended combination of oral and injectable medications, prescribed at the correct dose per weight. Guideline adherence for each outcome was coded as 0 (no) or 1 (yes).

#### Individual and site characteristics

Sociodemographic and clinical information was collected by patient interview and medical chart abstraction. Individual characteristics included age, sex, history of TB disease, HIV status, and weight. Additionally, anti-retroviral treatment status, prior household TB exposure, education level, relationship status, and employment status were captured to further describe the sample. Two site characteristics were examined: (1) the parent study categorized each site as rural or urban (defined as urban/peri-urban) and (2) geographic location, defined by treatment site province as Eastern Cape (EC) or KwaZulu Natal (KZN).

Rural / urban classification was determined by proximity to a metropolitan area and the population size of that hospital. The parent study further sub-divides the data into 3 categories, urban, peri-urban, and rural consistent with the South African National Statistics Office known as Statistics SA [17]. Further, each hospital CEO confirmed the designation of the hospital site as categorized in the parent study.

### Data analysis

Descriptive statistics were used to summarize (a) demographic and clinical characteristics of patients; (b) site characteristics of the healthcare system; and (c) guideline adherence outcomes, defined as whether the correct regimen and subcomponents were prescribed as per guideline for each patient at treatment initiation. Non-directional statistical tests were performed with the level of significant set at 0.05 for all tests. Data were cleaned and missing values were entered by verification on scanned case report forms.

#### Guideline concordance

The number (n) and percent (%) of patients prescribed the following per guidelines were determined for: (a) medications; (b) medications and doses (dose above or below a weight band); and (c) *regimen* (correct medications, doses, and frequencies). We also examined the data by (a) oral and (b) injectable medications, and (c) treatment site.

Bivariate logistic regression was used to test whether each individual and site characteristic predicted the guideline adherence outcomes. Characteristics evaluated were age (oldest to youngest), sex (males/females), history of TB disease (yes/no) disease, HIV coinfection(yes/no), urban site (urban/rural), and EC province site (EC/KZN). For each guideline outcome, a characteristic that significantly predicted the guideline outcome at the 0.10 level in the bivariate regression model was included in multivariable logistic regression model. Each multivariable model was then reduced using an interactive backward elimination process to remove the least significant predictor until a final model that included only significant predictor at the 0.05 level. To address effect size and clinical significance, the odds ratio (OR) and 95% confidence intervals for each explanatory variable was calculated.

## Results

### Sample characteristics

The analysis sample was comprised of 337 patients enrolled in the parent study and treated for DR-TB in South Africa. The mean age was 34.8 years (range: 15 to 75 years), with 19% youth, 53% male, 5% with no formal education, 64% unemployed, 73% HIV coinfected, and 51% with prior history of TB disease (Table 1). Three of the 10 sites were urban/peri-urban, while seven were rural. All three sites located in the Eastern Cape (EC) were rural. Among the 337 patients, 257 (76%) were treated at a rural site and159 (47%) were treated in the EC.

**Table 1:** Sample characteristics for patients with DR-TB.

Baseline Characteristic	N	Total (N = 337)
Age, in years, *mean* ± *SD*	337	34.8 ± 11.1
Youth (15–24 years of age), *n (%)*	337	64 (18.9)
Male sex, *n (%)*	337	179 (53.1)
Education level, *n (%)*	333	
None		15 (4.5)
Some education		294 (88.3)
Attended university		24 (7.2)
Employment status, *n (%)*	329	
Unemployed		183 (55.6)
Employed		120 (36.5)
Student		26 (7.9)
Living with partner, *n (%)*	337	63 (18.7)
Eastern Cape (EC) province site, *n (%)*	337	159 (47.2)
Rural site, *n (%)*	337	257 (76.3)
HIV coinfection, *n (%)*	332	243 (73.2)
HIV patients on ART	157	135 (86.0)
History of TB treatment, *n (%)*	332	169 (50.9)
Prior household TB exposure, *n (%)*	317	85 (26.8)
Household exposure to DR-TB, *n (%)*	73	32 (43.8)
Weight category, *n (%)*	337	
Group 1 (<33kg)		2 (0.59)
Group 2 (33-50kg)		104 (30.9)
Group 3 (51-70kg)		188 (55.8)
Group 4 (>70kg)		43 (12.8)

Attended university includes: any university, completed university; Employed includes: full time, part time, homemaker, and retired; Living with partner includes: married, living with a partner. ART = anti-retroviral treatment; Among the 243 with HIV co-infection, only 157 had ART information available.

#### Guideline adherence

Table 2 presents the overall guideline adherence rates. Among 337 patients, 295 (88%) were correctly prescribed all five medications. Of those 295, 23 patients did not have a dosage recorded for at least one of five medications. Thus, 314 patients were evaluated for (1) medications and doses and (2) *regimen*. Among 314, 103 (33%) were prescribed the correct medications and dosages, and 95 (30%) were prescribed correct *regimen*.

**Table 2:** Guideline adherence: Medications, medications and doses, and regimen.

Guideline	N	All Four Oral Medications Prescribed (n, %)	Any Injectable Medication Prescribed (n, %)	All Four Oral and Any Injectable Medications Prescribed (n, %)
Medications	337	306 (90.8)	325 (96.4)	295 (87.5)
Medications and Doses	314	128 (40.8)	166 (52.9)	103 (32.8)
Regimen (medications, doses, frequencies)	314	99 (31.5)	97 (30.9)	95 (30.3)

Four oral medications include: Moxifloxacin, Ethionamide, Terizidone, and Pyrazinamide. Injectable medications include: Kanamycin, Capreomycin, or Amikacin. Doses for each medication per weight are described in S1 Table.

There was site variation in the adherence outcomes. Medication adherence per site ranged from 63% to 100%, while correct medication and dose rates per site ranged from 0% to 80%. The *regimen* adherence rate was 0% to 70%, with only one site greater than 50% (Table 3).

**Table 3:** Site, individual characteristics, and treatment as per guideline by site.

**Site Characteristics**												
Patients treated (n, %)	**337**	**6** (1·8)	**50** (14·8)	**24** (7·1)	**10** (3·0)	**25** (7·4)	**51** (15·1)		**11** (3·3)	**60** (17·8)	**83** (24·6)	**17** (5·0)
Urban/Rural		U	U	U	R	R	R		R	R	R	R
Province		KZN	KZN	KZN	KZN	EC	EC		KZN	KZN	EC	KZN
**Patient Characteristics**												
Age, in years	337	38·0 (9·6)	31·7 (8·8)	39·2 (12·9)	33·6 (10·2)	43·9 (14·7)	35·1 (11·3)		34·4 (14·2)	35·8 (10·7)	32·5 (8·7)	31·6 (11·4)
Male sex (n, %)	337	5 (83·3)	29 (58·0)	15 (62·5)	5 (50·0)	13 (52·0)	29 (56·9)		3 (27·3)	27 (45·0)	42 (50·6)	11 (64·7)
HIV co-infection (n, %)	332	6 (100·0)	37 (74·0)	20 (83·3)	8 (80·0)	20 (80·0)	31 (64·6)		8 (72·7)	47 (79·7)	54 (65·9)	12 (70·6)
History of TB disease (n, %)	332	3 (50·0)	16 (32·0)	13 (56·5)	5 (50·0)	17 (68·0)	20 (39·2)		3 (50·0)	29 (58·9)	49 (59·8)	11 (64·7)
**Treatment as per South African DR-TB Guideline**												
**Sample size**^**1**^	**337**	**6**	**50**	**24**	**10**	**25**	**51**		**11**	**60**	**83**	**17**
Medications (n, %)		5 (83·3)	47 (94·0)	24 (100·0)	10 (100·0)	21 (84·0)	32 (62·8)		11 (100·0)	56 (93·3)	73 (88·0)	16 (94·1)
**Sample size**^**2**^	**314**	**6**	**40**	**16**	**10**	**25**	**51**		**10**	**56**	**83**	**17**
Medications & Doses (n, %)		0 (0·0)	13 (32·5)	2 (12·5)	8 (80·0)	10 (40·0)	15 (29·4)		5 (50·0)	19 (33·9)	27 (32·5)	4 (23·5)
Regimen (n, %)		0 (0·0)	10 (25·0)	2 (12·5)	7 (70·0)	10 (40·0)	15 (29·4)		5 (50·0)	15 (26·8)	27 (32·5)	4 (23·5)

Mean ± standard deviation (SD) for age; n (%) for categorical characteristics; Prescribed DR-TB treatment per guideline = n (%); U = Urban/peri-urban; R = Rural; KZN = KwaZulu Natal province; EC = Eastern Cape province; Regimen = medications, doses, and frequencies per guideline; Sample size^1^ = Number of patients with medication data available (N); Sample size^2^ = Number of patients with medication, dose, and frequency data available (N).

Among the 337 patients, 306 (91%) were correctly prescribed at least four oral medications: Moxifloxacin, Ethionamide, Pyrazinamide, and Terizidone and 325 (96%) were correctly prescribed one injectable medications: Kanamycin, Amikacin, or Capreomycin. Among the oral medications, Moxifloxacin, Pyrazinamide, and Terizidone were each prescribed for 99% of the patients, while Ethionamide was prescribed in 92%. The most commonly prescribed injectable medication was Kanamycin (73%), while Capreomycin was rarely prescribed (< 1%).

*Guideline adherence*: *Relation to individual and site characteristics*

Table 4 presents the univariate regression results for the adherence outcomes. Age, HIV coinfection, urban site, and EC province were significant predictors of whether correct medications were prescribed (Table 4). The odds of medication adherence were higher in patients who were younger, HIV coinfected, treated in a rural site, and seen at sites in the KZN province. None of the characteristics significantly predicted correct (1) medications and doses or (2) *regimen*.

**Table 4:** Guideline adherence: Univariate analysis.

Outcome	*N*	Wald chi-square	OR	95% CI	*p*-value
**Medications**					
Age	337	5·439	0·969	0·943–0·995	0·020
Sex	337	0·787	1·346	0·698–2·598	0·375
History of TB disease	332	0·084	1·102	0·573–2·119	0·772
HIV coinfection	332	4·953	2·155	1·096–4·236	0·026
Urban site	337	9·096	0·355	0·181–0·696	0·003
EC Province site	337	16·342	0·203	0·094–0·440	<·001
**Medications and Doses**					
Age	314	0·142	1·004	0·983–1·025	0·706
Sex	314	0·204	0·897	0·559–1·439	0·652
History of TB disease	309	0·583	0·831	0·517–1·336	0·445
HIV coinfection	309	1·571	0·715	0·423–1·208	0·210
Urban site	314	0·432	1·173	0·729–1·888	0·511
EC Province site	314	0·001	0·991	0·619–1·588	0·970
**Regimen**					
Age	314	0·065	0·997	0·976–1·019	0·798
Sex	314	0·262	0·882	0·544–1·429	0·609
History of TB disease	309	0·574	0·829	0·510–1·347	0·449
HIV coinfection	309	2·556	0·648	0·381–1·103	0·110
Urban Site	314	0·264	1·136	0·698–1·847	0·608
EC Province site	314	0·914	1·266	0·781–2·052	0·339

Table 5 presents the multivariable regression model for the medication adherence outcome. After covarying for the effects of other predictors, the results of the multivariable model confirmed the results of the bivariate models. Patients who were (1) younger, (2) HIV coinfected; (3) treated at rural sites; and (4) treated in KZN province were more likely to have correct medications prescribed.

**Table 5:** Guideline medication adherence: Multivariable logistic regression results.

Predictors	Wald chi-square	aOR	95% CI	*p*-value
Age	4·134	0·971	0·944–0·999	0·042
HIV coinfection	3·997	2·089	1·015–4·303	0·046
No HIV-coinfection *(ref)*				
Urban site	5·660	0·421	0·206–0·859	0·017
Rural site *(ref)*				
EC province site	12·507	0·240	0·109–0·529	0·001
KZN province *(ref)*				

N = 332; aOR = adjusted odds ratio; 95% CI = 95% confidence interval; EC = Eastern Cape; KZN = KwaZulu Natal; age organized in descending order; ref = reference group

#### Over and under dosing

Dosing was explored by medication and weight group to understand over and under dosing, independent of other medications (Table 6). Moxifloxacin was prescribed correctly to over 99% of participants, while Kanamycin was correctly prescribed to 60% of participants. Over dosing occurred in 0% (Amikacin) to 7% (Ethionamide) of participants. Under dosing occurred between 0% (Moxifloxacin) and 34% (Amikacin) with Pyrazinamide under dosed 30% and Kanamycin under dosed 34% of the time. Thirty medications were over dosed, while 285 were under dosed.

**Table 6:** Over and under dosing for standardized DR-TB medications.

Medications	*N*	Correct Dose n (%)	Over Dose n (%)	Under Dose n (%)	Missing Dose n (%)
Moxifloxacin	334	333 (99·7%)	1 (0·3%)	**-**	**-**
Weight Group 1		2 (100·0%)	-	-	-
Weight Group 2		101 (99·0%)	1 (0·3%)	-	-
Weight Group 3		187 (100·0%)	-	-	-
Weight Group 4		43 (100·0%)	-	-	-
Ethionamide	310	255 (82·3%)	22 (7·1%)	32 (10·3%)	1 (0·3%)
Weight Group 1		2 (100·0%)	-	-	-
Weight Group 2		69 (71·9%)	22 (22·9%)	4 (4·2%)	1 (1·0%)
Weight Group 3		149 (86·1%)	-	24 (13·9%)	-
Weight Group 4		35 (89·7%)	-	4 (10·3%)	-
Pyrazinamide	336	222 (66·1%)	1 (0·3%)	102 (30·4%)	11 (3·3%)
Weight Group 1		2 (100·0%)	-	-	-
Weight Group 2		98 (95·2%)	-	1 (1·0%)	4 (3·9%)
Weight Group 3		90 (47·9%)	1 (0·5%)	90 (47·9%)	7 (3·7%)
Weight Group 4		32 (74·4%)	-	11 (25·6%)	-
Terizidone	335	292 (87·2%)	1 (0·3%)	42 (12·5%)	-
Weight Group 1		2 (100·0%)	-	-	-
Weight Group 2		66 (64·1%)	-	37 (35·9%)	-
Weight Group 3		183 (97·9%)	1 (0·5%)	3 (1·6%)	-
Weight Group 4		41 (95·4%)	-	2 (4·7%)	-
Kanamycin	245	147 (60·0%)	5 (2·0%)	82 (33·5%)	11 (4·5%)
Weight Group 1		1 (100·0%)	-	-	-
Weight Group 2		59 (83·1%)	5 (7·0%)	-	7 (9·9%)
Weight Group 3		56 (40·0%)	-	80 (57·1%)	4 (2·9%)
Weight Group 4		31 (93·9%)	-	2 (6·1%)	-
Amikacin	79	52 (65·8%)	-	27 (34·2%)	-
Weight Group 1		1 (100·0%)	-	-	-
Weight Group 2		23 (76·7%)	-	7 (23·3%)	-
Weight Group 3		23 (56·1%)	-	18 (43·9%)	-
Weight Group 4		5 (71·4%)	-	2 (28·6%)	-

Weight group 1 ≤ 33kg; weight group 2 = 33-50kg; weight group 3 = 51-70kg; weight group 4 ≥ 70kg.

## Discussion

Most patients received the correct five standardized medications, yet only 30% received guideline adherent *regimens* (medications, dosages, and frequencies). Younger patients, those HIV coinfected, receiving care at rural sites, and living in KZN were more likely to have the correct medication prescribed. None of the factors were associated with correct prescription of medications and dosage, or full *regimen*.

With each additional year of age, patients were 3% less likely to be prescribed correct medications. Clinicians may be wary of prescribing certain medications in older patients due to risk for toxicities and thus, could exclude recommended medication. South African guidelines quote anecdotal evidence that adolescents are at high risk for poor treatment outcomes [5]. However, the analysis showed that younger patients were more likely to be prescribed correct medications. This did not hold true for (1) medications and dosages nor (2) *regimen*. The developmental stage youth are in is critical in establishing self-management and health-related behaviors [18]. This analysis only included 64 youth (15–24 years), so including adolescents in future DR-TB studies is warranted. Additionally, more research in older adult prescribing could provide insight into actual versus perceived toxicities.

Patients with HIV coinfection were twice as likely to be prescribed correct medications than those without HIV. Clinicians may be more vigilant to correctly prescribe to those with HIV. Patients at rural sites were nearly two and one half times more likely to receive correct medications than those in urban areas and patients in KZN had over four times a greater likelihood of being prescribed correct medications than patients receiving care in the EC.

Although patients treated at rural sites were more likely to have the correct medication prescribed according the guidelines, the findings indicate that other factors not measured within each province are also likely to significantly influence the medication guideline adherence. More specifically, the patients treated in the KZN province which is comprised of a mixture of rural and urban sites were more likely to have the correct medication prescribed than patients treated in the Eastern Cape province which included only rural sites. These findings indicate that rural/urban setting within a province is not the only factor influence guideline adherence. The Eastern Cape province has the lowest gross domestic product per capita, and may be a reason for less adherence by healthcare workers due to lack of physical resources, training, or human resources [19]. Additionally, variation in provincial healthcare resourcing may have influenced province outcome (EC versus KZN) [20].

Ensuring appropriate use of standard regimens is one solution to managing DR-TB [5, 21]. However, infrastructure in South Africa may not adequately support systematic guideline-based care. Clinician adherence to TB treatment prescribing has been shown to vary in South Africa and other countries [22, 23]. One systematic review described health care workers’ lack of TB regimen knowledge, ranging between 8% and 93% [24]. Another systematic review described 67% of treatment regimens as inappropriate across 37 studies in 22 countries [25]. This study adds to TB treatment literature because, to our knowledge, no other studies have evaluated DR-TB treatment regimens inclusive of medications, dosages, and frequencies in South Africa.

Toxicities are common with DR-TB medications [26]. Despite these risks, under dosing threatens the ability to fully treat individuals, and can lead to DR-TB transmission. Pyrazinamide was commonly under dosed which is an important part of DR-TB treatment as it has bactericidal activity to semi dormant mycobacteria and Pyrazinamide resistance has been reported in nearly 50% of some South African studies [27]. Pyrazinamide is frequently given as part of first-line TB treatment, raising concern for developed resistance in patients previously treated for drug-susceptible TB. Fluoroquinolones are also an important class of medication for individuals with DR-TB as their use has shown to improve survival [21]. Thus, under dosing medications that are cornerstones of DR-TB regimens is of great concern.

Under-dosing could also be related to pill formulations, making accurate dosing according to guidelines difficult. For example, in South Africa Pyrazinamide only is available in 500mg tablets. Many countries provide fixed dose combination (FDC) therapy for drug susceptible TB to improve patient acceptance and to decrease pill burden [28]. However, no FDC therapy exists currently for DR-TB. When standardized treatments are consistently used they can decrease errors in prescribing, maintain drug supply and facilitate procurement processes, and reduce costs [28].

Changing provider behavior and implementing policies is difficult. These findings are important due to South Africa expanding prescriptive practice to trained DR-TB nurses and guidelines moving towards shorter regimens, yet increasing the number of medications prescribed [5, 29]. Ensuring guidelines are effectively implemented is critical, and next steps include understanding the reasons for poor guideline adherence.

### Limitations

We conservatively excluded 204 individuals due to laboratory and medical history results that could have affected prescribed treatment regimens. However, many diagnostic results are typically not available to prescribing clinicians at treatment initiation (i.e. labs were drawn on the same day as initiation). Thus, in most clinical settings, it is possible that the recommended standard treatment at the time of initiation would have been viewed as appropriate for the 204 patients excluded from this analysis. The cautious approach to exclude the 204 patients from this secondary analysis resulted in a smaller sample size with less statistical power and external validity, we felt this approach was warranted because the known laboratory and medical history results could have led to deviations from guideline adherence. While the availability of additional drug sensitivity tests or line probe assay results may have been possible, the parent study from which this data was drawn focused on individuals with rifampicin resistant TB and excluded anyone with known pre-XDR and XDR-TB infection at initiation. As such, we believe the availability of additional DST information, which would influence a prescriber to individualize a treatment regimen, was limited. Second, regimen was assessed independent of treatment timing. Timely initiation is important in addition to correct regimen prescription [30]. Third, frequencies may have been negatively biased in the *regimen* outcome as only individuals with correct dosages were analyzed for frequency. Finally, these findings are mostly descriptive and did not consider root causes for prescribing deviations–standardized treatment may not be appropriate for every individual. Despite these limitations, this study enriches the literature regarding provider adherence to DR-TB guidelines in low-resource settings.

### Conclusion

There is poor adherence to national DR-TB guidelines in South Africa with only 30% of patients being prescribed the correct *regimen* at treatment initiation. Although most individuals with DR-TB are prescribed correct medications, few are prescribed correct doses for all medications. Under-dosing is more common than over dosing, which could lead to increased drug resistance. Designing interventions to facilitate appropriate prescribing and enhancing providers’ ability to prescribe effective DR-TB treatment can improve patient outcomes and prevent transmission.

## Supporting information

S1 Table Standardized DR-TB regimen for adults and children 8 years and older. (DOCX)

S1 File Dataset. (SAS7BDAT)

S2 File Data dictionary. (DOCX)
