# Supplementary material for: Provision of guideline-based care for drug-resistant tuberculosis in South Africa: Level of concordance between prescribing practices and guidelines
Source: PLoS One. 2018 Nov 5;13(11):e0203749. doi: 10.1371/journal.pone.0203749 (PMC6218024; doi:10.1371/journal.pone.0203749)
Supplement: S1 Table — (DOCX) [file pone.0203749.s001.docx]

**S1 Table. Standardized DR-TB regimen for adults and children 8 years and older**

| **Weight Group** | **Patient Weight** | **Medication^1^** | **Dosage** |
| --- | --- | --- | --- |
| 1 | < 33 kg | Moxifloxacin | 400 mg |
|  |  | Ethionamide | 15-20 mg/kg |
|  |  | Pyrazinamide | 30-40 mg/kg |
|  |  | Terizidone | 15-20 mg/kg |
|  |  | Kanamycin,  Amikacin, or  Capreomycin | 15-20 mg/kg |
| 2 | 33-50 kg | Moxifloxacin | 400 mg |
|  |  | Ethionamide | 500 mg |
|  |  | Pyrazinamide | 750 mg |
|  |  | Terizidone | 750 mg |
|  |  | Kanamycin,  Amikacin, or  Capreomycin | 500-750 mg,  750-1000 mg,  500-750 mg |
| 3 | 51-70kg | Moxifloxacin | 400 mg |
|  |  | Ethionamide | 750 mg |
|  |  | Pyrazinamide | 1750-2000 mg |
|  |  | Terizidone | 750 mg |
|  |  | Kanamycin,  Amikacin, or  Capreomycin | 1000 mg |
| 4 | > 70 kg | Moxifloxacin | 400 mg |
|  |  | Ethionamide | 750-1000 mg |
|  |  | Pyrazinamide | 2000-2500 mg |
|  |  | Terizidone | 750-1000mg |
|  |  | Kanamycin,  Amikacin, or  Capreomycin | 1000 mg |

Developed from Table XVI Intensive Phase: Standardized Regimen for Adult and Children 8 Years and Older (MDR-TB Treatment) from Department of Health: Republic of South Africa. (2013). *Management of Drug-Resistant Tuberculosis*. Pretoria: Republic of South Africa (p. 48). ^1^All oral medications are required to be prescribed at least six days per week; any injectable (Kanamycin, Amikacin, and Capreomycin) are required to be prescribed at least five days per week.
