# Supplementary material for: Provision of guideline-based care for drug-resistant tuberculosis in South Africa: Level of concordance between prescribing practices and guidelines
Source: PLoS One. 2018 Nov 5;13(11):e0203749. doi: 10.1371/journal.pone.0203749 (PMC6218024; doi:10.1371/journal.pone.0203749)
Supplement: S2 File — (DOCX) [file pone.0203749.s003.docx]

Data dictionary for Plos One Global Health manuscript

“**Provision of guideline-based care for drug-resistant tuberculosis in South Africa: Level of concordance between prescribing practices and guidelines**”

The dataset  is for 337 participants.

The dataset has 1930 records because this is stacked dataset in which there is one record per patient per medication. Thus, there may multiple records per patient if the patient was prescribed more than one TB medication.

The value for the dose-by-weight variable is for medication indicated by the med_full_name variable.

The dataset is sorted by patient (record_id) and medication full name (med_full_name).

For each patient, the other information (variables and values) on each record is the same.

| Variable Name | Variable Type | Coded Values | Variable Definition |
| --- | --- | --- | --- |
| Record_id | Integer |  | Patient record ID |
| Med_full_name | Short text | Amikacin  Capreomycin  Ethambutol  Ethionamide  Isoniazid  Kanamycin  Linezolid  Moxifoxaci  Other  Pyrazinamid  Terizidone | DR-TB drugs |
| Age_final | numerical | 15.2-74.9 | Patient age |
| Youth | 0,1 |  | 0=25+, 1=13-24 |
| Intervention_site | 0,1 |  | 0=no; 1=yes |
| EC_province | 0,1 |  | 0=No, 1=Yes |
| Female_sex | 0,1 |  | 0=No, 1=Yes |
| TB_prior2 |  |  | 0=No, 1=Yes |
| Hiv2 | 0,1 |  | 0=reactive, 1 = non-reactive |
| Hiv_art | Short text |  | No, yes |
| TTT_adj_final | Integer | 0-142 days | Time to treatment |
| First_med_day_range | Integer | -5-16 days | Range between first medication and other medications prescribed |
| Demo_educ_level | Short text | None  Some primary school, not complete  Primary school, complete  Some secondary school, not complete  Secondary school, complete  Some technical school, not complete  Technical school, complete  Some college or university, not complete  College or University, complete  Other | What is your highest level of education |
| Demo_employ_status | Short text | Unemployed  Homemaker  Employed; Full-time  Employed: Part-time/Seasonal  Retired/pentioner  Student | Before becoming a patient, what best described your employment status |
| Demo_marital_stat | Short text | Single  Married  Separated/Divorced  Widowed  Living with girlfriend/boyfriend, but not married  In a relationship with a girlfriend/boyfriend, but not living together | Marital status: Are you now… |
| Amikacin | 0,1 |  | 0=not prescribed, 1=prescribed |
| Capreomycin | 0,1 |  | 0=not prescribed, 1=prescribed |
| Ethionamide | 0,1 |  | 0=not prescribed, 1=prescribed |
| Kanamycin | 0,1 |  | 0=not prescribed, 1=prescribed |
| Moxifloxicin | 0,1 |  | 0=not prescribed, 1=prescribed |
| Pyrazinamide | 0,1 |  | 0=not prescribed, 1=prescribed |
| Terizidone | 0,1 |  | 0=not prescribed, 1=prescribed |
| Am_dose | 0,1 |  | 0=not correct dose, 1=correct dose |
| Am_freq | 0,1 |  | 0=not correct frequency,  1= correct frequency |
| Am_given | 0,1 |  | 0=not prescribed,  1=prescribed |
| Capreo_dose | 0,1 |  | 0=not correct dose, 1=correct dose |
| Capreo_freq | 0,1 |  | 0=not correct frequency,  1= correct frequency |
| Capreo_given | 0,1 |  | 0=not prescribed,  1=prescribed |
| Eto_dose | 0,1 |  | 0=not correct dose, 1=correct dose |
| Eto_freq | 0,1 |  | 0=not correct frequency,  1= correct frequency |
| Eto_given | 0,1 |  | 0=not prescribed,  1=prescribed |
| Km_dose | 0,1 |  | 0=not correct dose, 1=correct dose |
| Km_freq | 0,1 |  | 0=not correct frequency,  1= correct frequency |
| Km_given | 0,1 |  | 0=not prescribed,  1=prescribed |
| Moxi_dose | 0,1 |  | 0=not correct dose, 1=correct dose |
| Moxi_freq | 0,1 |  | 0=not correct frequency,  1= correct frequency |
| Moxi_given | 0,1 |  | 0=not prescribed,  1=prescribed |
| Pza_dose | 0,1 |  | 0=not correct dose, 1=correct dose |
| Pza_freq | 0,1 |  | 0=not correct frequency,  1= correct frequency |
| Pza_given | 0,1 |  | 0=not prescribed,  1=prescribed |
| Teri_dose | 0,1 |  | 0=not correct dose, 1=correct dose |
| Teri_freq | 0,1 |  | 0=not correct frequency,  1= correct frequency |
| Teri_given | 0,1 |  | 0=not prescribed,  1=prescribed |
| Ethambutol_dose | 0,1 |  | 0=not correct dose, 1=correct dose |
| Ethambutol_freq | 0,1 |  | 0=not correct frequency,  1= correct frequency |
| Ethambutol_given | 0,1 |  | 0=not prescribed,  1=prescribed |
| Correct_inject_meds | 0,1 |  | 0=incorrect; 1=correct |
| Correct_inect_meds_dose | 0,1,8,9 |  | 0=incorrect; 1=correct 8=missing; 9=not prescribed |
| Correct_inject_regimen | 0,1,9,88 |  | 0=incorrect; 1=correct; 9=not prescribed; 88=missing |
| Correct_meds | 0,1 |  | 0=incorrect meds; 1=correct meds |
| Correct_meds_dose | 0,1,8,9 |  | 0=incorrect dose; 1=correct dose; 8=missing; 9=not prescribed |
| Correct_oral_meds | 0,1 |  | 0=incorrect oral meds; 1=correct oral meds |
| Correct_oral_meds_dose | 0,1,8,9 |  | 0=incorrect dose; 1=correct dose; 8=missing; 9=not prescribed |
| Correct_oral_regimen | 0,1,9,88 |  | 0=incorrect; 1=correct; 9=not prescribed; 88=missing |
| Correct_regimen | 0,1,9,88 |  | 0=incorrect regimen; 1=correct regimen; 9=not prescribed; 88=missing |
| Inject_med | 0,1 | At least one injectable guideline medication name listed | 0=no, 1=yes |
| Oral_med | 0,1 | At least one oral guideline medication name listed | 0=no; 1=yes |
| Dose_by_weight | Numerical | 0.95-48.61 | Medication dose/weight ratio |
| Weight_group | Categorical | 1,2,3,4 | **1=(<33kg), 2=(33-50kg), 3=(51-70kg), 4=(>70kg)** |
|  |  |  |  |
|  |  |  |  |
|  |  |  |  |
|  |  |  |  |
|  |  |  |  |
|  |  |  |  |
|  |  |  |  |
